# Exercise as an Adjuvant Treatment of Schizophrenia: A Review

**DOI:** 10.7759/cureus.42084

**Published:** 2023-07-18

**Authors:** Muhammad S Abbas, Sondos T Nassar, Tasniem Tasha, Anjali Desai, Anjana Bajgain, Asna Ali, Chandrani Dutta, Khadija Pasha, Salomi Paul, Sathish Venugopal

**Affiliations:** 1 Psychiatry, California Institute of Behavioral Neurosciences & Psychology, Fairfield, USA; 2 Medicine and Surgery, California Institute of Behavioral Neurosciences & Psychology, Fairfield, USA; 3 Internal Medicine, California Institute of Behavioral Neurosciences & Psychology, Fairfield, USA; 4 Medicine, California Institute of Behavioral Neurosciences & Psychology, Fairfield, USA; 5 Division of Research & Academic Affairs, California Institute of Behavioral Neurosciences & Psychology, Fairfield, USA; 6 Family Medicine, California Institute of Behavioral Neurosciences & Psychology, Fairfield, USA; 7 Pediatrics, California Institute of Behavioral Neurosciences & Psychology, Fairfield, USA

**Keywords:** schizophrenia and other psychotic disorders, schizophrenia, management, treatment, physical acitivity, excercise

## Abstract

Schizophrenia is a chronic debilitating condition associated with impaired social functioning, memory, and executive functioning. To date, we are still unsure about the exact etiology of schizophrenia, but there are many factors, such as genetics, diminished hippocampal volume, and imbalance of neurotransmitters, that lead to the pathogenies of the disease. Antipsychotics are the most effective treatment option for schizophrenia so far, yet they are associated with a wide array of side effects, ranging from extrapyramidal side effects to conditions, such as metabolic syndrome. Exercise has been shown to increase neural connections in the brain, which can improve cognition and memory. This literature review focuses on the signs and symptoms of schizophrenia, its treatment options, and how exercise can help with some of the symptoms of schizophrenia, especially the negative symptoms that are least effectively treated by antipsychotics.

## Introduction and background

Schizophrenia is a chronic condition characterized by a contorted perception of reality characterized by overwhelming functional impairment. There are several psychiatric symptoms of schizophrenia, such as delusions, disorganized speech, disorganized or catatonic behavior, and negative symptoms. Most patients with schizophrenia have cognitive decline as a natural progression of the disease. Although the exact etiology of schizophrenia is unknown, it is known that alterations in the neurotransmitters and changes in the size of the hippocampus lead to the development of the disease [1]. In addition to pharmacological treatment, which is mainly restricted to a wide array of antipsychotic agents, other modalities, such as physical activity, have been proposed as adjuvant or primary treatment options. Numerous meta-analyses [2] and randomized controlled trials [3] have shown the positive effects of physical activity on the symptoms of schizophrenia. Antipsychotics, which is the mainstay treatment option for schizophrenia, are associated with many adverse effects, such as metabolic syndrome, weight gain, glucose disturbance, and lipid dysregulation. In fact, the prevalence of the metabolic syndrome is twice in patients being treated with antipsychotics [4], and physical activity proves to manage these adverse effects of antipsychotics. Physical activity is essential for health as it helps in improving the quality of life and upgrading our general health [5]. In addition, physical activity plays a vital role in relieving some of psychological disorders’ symptoms as it reinforces the neural connections that were once distorted and led to these psychological disorders in the first place, and this can lead to the enhancement of cognition and social functioning, domains that are very disabling for patients with schizophrenia [6].

This literature review primarily focuses on the etiology and symptoms of schizophrenia, the effects of physical activity on its symptoms, and the current understanding of exercise programs as an effective treatment of the disease. This would form a basis for understanding whether physical activity can act as an add-on to pharmacological treatment.

## Review

Methods

For this literature review, articles published after the year 1990 were selected by searching PubMed with regular keywords and major Medical Subject Heading (MeSH) keywords, Google Scholar, and Science Direct. Studies published after the year 1990, in the English language, and based on male and female human studies were selected. Exclusion criteria consist of literature in languages other than English and animal studies. Table 1 shows a detailed description of database search terms and their respective results.

**Table 1:** Summary of databases used, search strategy and keywords, inclusion criteria, and results MeSH: Medical Subject Headings

Database name	Search strategy and keywords	Inclusion criteria for the search filter	Results
PubMed	( "Schizophrenia/analysis"[Mesh] OR "Schizophrenia/anatomy and histology"[Mesh] OR "Schizophrenia/cerebrospinal fluid"[Mesh] OR "Schizophrenia/diagnosis"[Mesh] OR "Schizophrenia/diagnostic imaging"[Mesh] OR "Schizophrenia/drug effects"[Mesh] OR "Schizophrenia/drug therapy"[Mesh] OR "Schizophrenia/enzymology"[Mesh] OR "Schizophrenia/epidemiology"[Mesh] OR "Schizophrenia/etiology"[Mesh] OR "Schizophrenia/genetics"[Mesh] OR "Schizophrenia/history"[Mesh] OR "Schizophrenia/immunology"[Mesh] OR "Schizophrenia/pathology"[Mesh] OR "Schizophrenia/physiology"[Mesh] OR "Schizophrenia/physiopathology"[Mesh] OR "Schizophrenia/prevention and control"[Mesh] OR "Schizophrenia/rehabilitation"[Mesh] OR "Schizophrenia/statistics and numerical data"[Mesh] ) AND ( "Exercise/adverse effects"[Mesh] OR "Exercise/analysis"[Mesh] OR "Exercise/anatomy and histology"[Mesh] OR "Exercise/blood"[Mesh] OR "Exercise/economics"[Mesh] OR "Exercise/education"[Mesh] OR "Exercise/epidemiology"[Mesh] OR "Exercise/ethics"[Mesh] OR "Exercise/etiology"[Mesh] OR "Exercise/genetics"[Mesh] OR "Exercise/history"[Mesh] OR "Exercise/injuries"[Mesh] OR "Exercise/metabolism"[Mesh] OR "Exercise/pathology"[Mesh] OR "Exercise/psychology"[Mesh] OR "Exercise/therapeutic use"[Mesh] OR "Exercise/therapy"[Mesh] OR "Exercise/trends"[Mesh] OR "Exercise/veterinary"[Mesh] )	Sex: male and female; language: English; years: 1990-2022; full-text articles	64 results

Discussion

Diagnostic Criteria of Schizophrenia

According to the Diagnostic and Statistical Manual of Mental Disorders, Fifth Edition (DSM-5), a formal clinical diagnosis of schizophrenia can be made in a patient when two or more of the following five symptoms are present for a period of at least one month (one of the two must be symptom one, two, or three), and symptoms should last at least for six months: (1) delusions, (2) hallucinations, (3) disorganized speech, (4) disorganized or catatonic behavior, and (5) negative symptoms (affect flattening, anhedonia, alogia, and avolition). Symptoms of schizophrenia are divided into positive and negative symptoms. Positive symptoms are mainly hallucinations, delusions, disorganized behavior, and speech, whereas negative symptoms are flat affect (lack of emotional responsiveness), alogia (reduced speech), and anhedonia (lack of pleasure). [7] Cognitive deficits are widely recognized in schizophrenia although they are not included in the current International Classification of Diseases (ICD‐10) or DSM‐5 [8].

Etiology of Schizophrenia

Genetics: It is thought that genetic predisposition increases the risk of schizophrenia by up to 80%. Fraternal twins have a 17% chance, whereas identical twins have close to 48% chance of both developing schizophrenia if one twin develops the disease; if one parent has the disease, there is a 13% chance that their children might have it, and siblings have a probability of around 9% of developing the disease [9].

Neurotransmitter: The dopamine hypothesis, a theory about how dopamine imbalance leads to schizophrenia, proposes how excessive dopaminergic activity in the brain, especially in the mesolimbic pathway, leads to positive symptoms of schizophrenia. It also proposes that a decrease in dopaminergic activity in the prefrontal cortex causes negative symptoms [10]. Moreover, other neurotransmitters, such as norepinephrine (elevated levels), gamma-aminobutyric acid (GABA) (decreased levels), and serotonin (elevated levels), are involved in the pathogenesis of schizophrenia [11].

Structural Effects of Exercise on the Brain

**Figure 1:**
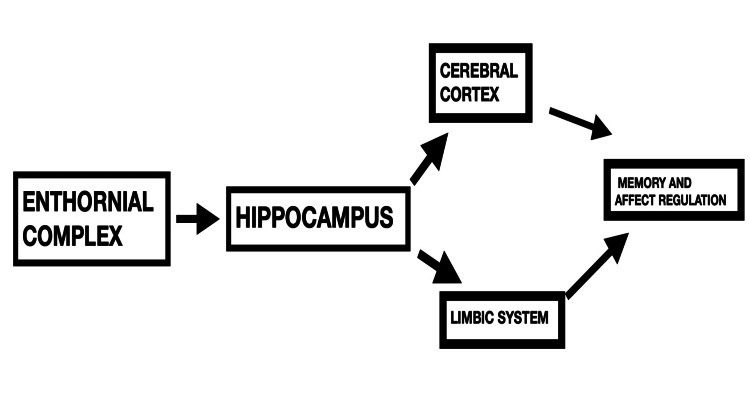
Summary of the pathway essential for mood and memory regulation.

According to FYSS (*Physical Activity in the Prevention and Treatment of Disease*), “physical activity is defined purely physiologically, as all body movements that increases energy use beyond resting levels.” There has been a lot of interest in employing aerobic physical activities to target symptoms of schizophrenia, as evidenced by the increasing number of publications in recent years [12]. Exercise is associated with an increase in brain volumes, and that is due to neurogenesis, which leads to procognitive effects as neurocognition is significantly impaired in patients with schizophrenia. [13] In healthy individuals, exercise has been shown to increase memory, brain processing power, and executive functioning [14]. These domains are usually impaired in people with schizophrenia as it leads to a decline in cognitive functioning, usually reported as being significantly lower than the general population, and these symptoms are very disabling for the patients [15]. The hippocampus, a primary center in the brain, is responsible for learning and memory, and it is known as a key player in the pathogenies of schizophrenia [16]. The hippocampus receives highly processed information from the enthornial complex (a part of the temporal lobe, which functions as a network hub for memory), and then this highly processed information is projected to the cortex and structures of the limbic system. This is crucial for two important brain functions, namely, memory and affect regulation. Both of these functions are abnormal in patients with schizophrenia [17]. Figure 1 shows a summary of how mood and memory are regulated.

**Figure 2:**
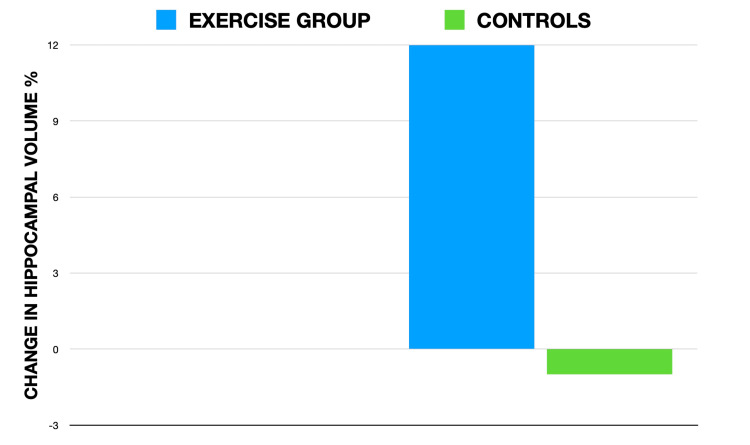
Results of the randomized controlled trial conducted by Pajonk et al.

The hippocampus also has a high degree of neuroplasticity, and in patients with schizophrenia, neural plasticity and adult neurogenesis are impaired [18]. All in all, these hippocampal abnormalities lead to deficits in memory, cognition, and executive functioning in schizophrenia [19]. Apart from this, schizophrenia is also associated with reduction of certain neurotropic peptides, such as brain-derived neurotrophic factor (BDNF) that helps in the neurogenesis and formation of synapses for memory, learning, and executive functioning [20]. Pharmacological treatment has no effect on neurogenesis and hippocampal volume [21]. In the randomized controlled trial conducted by Pajonk et al., it was demonstrated that three months of aerobic exercise training increased the hippocampal volume by 12% compared to the non-exercise group (-1%). Exercise also improved short-term memory, demonstrated by a 34% increase in short-term memory scores compared to those of controls. Figure 2 summarizes the results of the randomized controlled trial conducted by Pajonk et al. [22]. 

Generally, in people without schizophrenia, exercise is associated with increased brain processing and improved executive functioning and memory, and similar improvements were also seen in people with schizophrenia [23]. In summary, exercise leads to increased hippocampal volume, increased blood supply to the brain [24], and improvement in BDNF levels that lead to axonal and dendritic remodeling. Overall, exercise increases the cognition needed for daily life functioning, an aspect very important for patients with schizophrenia [25].

Another randomized controlled trial showed that exercise leads to increased white matter in fiber tracts involved with attention, memory, and executive function [26]. 

Explained above are the proposed mechanisms through which exercise leads to structural changes in the brain that lead to the improvement of symptoms. 

Effects of Exercise on the Symptoms of Schizophrenia

The positive symptoms of schizophrenia can be successfully managed by antipsychotics, yet the negative symptoms are harder to manage. Furthermore, these negative symptoms lead to poor functional outcomes in patients with schizophrenia [27]. In a large-scale meta-analysis, it was shown that exercise leads to a reduction in the total symptom severity and hence improvement in both negative and positive symptoms [28]. Other studies were also coherent in showing a reduction of symptoms in patients with schizophrenia, such as Sheewe et al. in his randomized controlled trial, which demonstrated a significant reduction of symptoms of schizophrenia compared to occupational therapy. Symptoms were reported based on the Positive and Negative Syndrome Scale (PANSS) [29].

Effects of exercise on the cognitive symptoms of schizophrenia: Cognitive impairment is very debilitating in patients with schizophrenia, and to date, clinicians are still very much in the dark about its effective management. It has been reported in a meta-analysis that exercise led to significant improvements in the global cognition of patients with schizophrenia, specifically in the subset where physical activities were supervised by professionals. The mechanism behind this improvement has been hypothesized to be due to increased serum levels of BDNF [30].

Effects of exercise on the negative symptoms of schizophrenia: Schizophrenia displays significant deficits in social functioning [31]. Poor social functioning represents a public health concern [32]. Interestingly, several therapeutic approaches, such as cognitive remediation, third-wave therapy, cognitive behavioral therapy (CBT), and pharmacotherapy, have been employed but showed controversial efficacy. In the following analysis, it was reported that exercise had substantial effects on social cognition. In addition, it has also been linked to increased employment, increased social functioning, and the ability to live independently [33].

Antipsychotic medications are associated with significant side effects, such as metabolic syndrome, obesity, and a shorter life span compared to the general population [34], and numerous studies have shown that the medication nonadherence to antipsychotics is around 41%. Obesity in particular was associated with impairment in self-esteem and distress related to physical appearance. Exercise can help mitigate and also address the psychological stress associated with the side effects of antipsychotic medications. Not restricted to schizophrenia, exercise has been shown to reduce symptoms in patients of depression. Blumenthal et al. compared the efficacy of exercise to antidepressant medication in reducing the clinical symptoms of depression. The randomized controlled trial was able to demonstrate exercise is as effective as medication in mitigating the symptoms of depression and also maintaining remission [35]. Similar results with a mean decrease by 6.46 points on the Beck Depression Inventory Scale in the exercise group were seen in the meta-analysis of a randomized controlled trial conducted by Schuch et al., showing how effective exercise can be in tackling symptoms of depression [36]. The pathogenesis of depression is similar to that of schizophrenia, with similarities, such as reduction in the hippocampal volume and reduction of synaptic plasticity being some of the major causes. Exercise has been shown to increase the levels of BDNF, which leads to an increase in neural connections in the brain, resulting in increased hippocampal volume and an increase in synaptic plasticity. The effects of exercise on schizophrenia as mentioned in different published literature works is summarized in Table 2.

**Table 2:** Summary of different literature results on the effects of exercise on symptoms of schizophrenia.

Effects of exercise on schizophrenia	Published literature
Improvement in depressive symptoms	Schuch et al. [36]
Improvement in total symptom severity	Dauwan et al. [27]
Improvement in memory and executive functioning	Smit et al. [14]
Improved cognition and social functioning	Kimhy et al. [6]
Improved social functioning and global cognition	Firth et al. [29]
Increase in white matter tracts resulting in improved processing speed, memory, and executive functioning	Svatkova et al. [25]
Improvement in short-term memory	Pajonk et al. [22]
Increase in neurotropic peptides, such as brain-derived neurotrophic factor	Svatkova et al. [25]
Increased neurogenesis	Erickson et al. [23
Improved blood flow and increased hippocampal volume	Pajonk et al. [22]

Limitations 

There are only limited studies in the literature that actually focus on exercise and its therapeutic effect on patients with schizophrenia. With the rising disease burden of schizophrenia worldwide, more resources need to be allocated to study adjuvant treatments, such as exercise, which has countless other benefits on the physical wellbeing and can save patients from the debilitating side effects of antipsychotics. With exercise, the biggest limitation is patient adherence. Patients with schizophrenia often struggle with motivation and the need to maintain consistent routines, especially in patients with reduced cognition, where the ability to understand instructions is a problem. Thus, additional research would be needed to better understand the most effective approach with regard to exercise frequency, duration, and a closer look at regular follow-up after such an intervention. Moreover, individual preference with regard to exercise programs increases adherence; thus, it may be a challenge to find effective and safe activities that are engaging and enjoyable for patients with schizophrenia as interests can vary widely. There is also more need to address safety concerns with regard to exercise programs for patients with schizophrenia. Impaired judgement and impulsivity serve as major risks, so there is a need to come up with exercise programs and equipment that tailor specifically to ensure safety of patients with schizophrenia, caregivers, and family members.

## Conclusions

Schizophrenia is a lifelong debilitating condition. Even though pharmacological therapy helps in managing the disease to a large extent, it is associated with side effects, such as glucose imbalance, obesity, and metabolic syndrome. Furthermore, among the debilitating symptoms of schizophrenia are cognitive dysfunction and other negative symptoms, against which antipsychotics are not that effective. Furthermore, side effects, such as weight gain and obesity, are associated with impairments in self-esteem and distress related to physical appearance. Physical activity has been shown to increase the hippocampal volume and levels of BDNF, which lead to improvements in cognition, functionality, and debilitating negative symptoms. Furthermore, physical activity can be helpful in mitigating the metabolic syndrome-related side effects of antipsychotics. While exercise can be a valuable addition to treat schizophrenia, it is very important to consider and address limitations that can maximize its benefits.
